# Structural Elucidation of the Lipooligosaccharide from the Deep-Sea Bacterium *Rheinheimera pacifica* KMM 1406^T^

**DOI:** 10.3390/md24090322

**Published:** 2026-09-15

**Authors:** Vlada S. Belova, Lyudmila A. Romanenko, Pavel S. Dmitrenok, Maxim S. Kokoulin

**Affiliations:** G.B. Elyakov Pacific Institute of Bioorganic Chemistry, Far Eastern Branch, Russian Academy of Sciences, 159/2, Prospect 100 let Vladivostoku, Vladivostok 690022, Russia; vladabelova306@gmail.com (V.S.B.); lro@piboc.dvo.ru (L.A.R.); paveldmt@piboc.dvo.ru (P.S.D.)

**Keywords:** *Rheinheimera pacifica*, deep-sea bacteria, lipooligosaccharide, lipid A, core oligosaccharide, structure, NMR spectroscopy, MALDI-TOF mass spectrometry

## Abstract

The chemical structure of the lipooligosaccharide (LOS) from the marine bacterium *Rheinheimera pacifica* KMM 1406^T^ was investigated. Analysis by SDS-PAGE with silver staining revealed a low-molecular-weight banding pattern characteristic of R-type lipopolysaccharide (LOS). The fatty acid composition was determined by GC-MS, revealing mainly dodecanoic (12:0), 3-hydroxydodecanoic [12:0(3-OH)], and 3-hydroxytetradecanoic [14:0(3-OH)] acids. The oligosaccharide (OS) was obtained by complete deacylation of the LOS. Monosaccharide analysis indicated the presence of D-GlcN, D-Gal, D-Glc, and a phosphorylated Kdo residue. Using NMR spectroscopy, the structure of the OS was established as: α-D-Gal*p*-(1→2)-α-D-Gal*p*-(1→6)-α-D-Glc*p*-(1→5)-α-Kdo*p*4P-(2→6)-β-D-Glc*p*N4P-(1→6)-α-D-Glc*p*N1P. Negative-ion MALDI-TOF mass spectrometry of the lipid A domain demonstrated the presence of bisphosphorylated penta-acylated and tetra-acylated molecular species. Tandem mass spectrometry determined the distribution of the acyl chains on the glucosamine disaccharide backbone. The LOS is characterized by a short carbohydrate chain and a low acylation degree of the lipid A domain.

## 1. Introduction

Structural adaptations of bacterial cell-surface components are crucial for the survival of microorganisms inhabiting deep-sea biotopes. Extreme marine environments, characterized by low ambient temperatures, high hydrostatic pressure, elevated salinity, and severely limited nutrient availability, demand specialized physiological mechanisms from their bacterial communities [1,2,3,4]. Among the cell envelope structures critical for enduring such abiotic stress, the lipopolysaccharides (LPSs) of Gram-negative bacteria are of paramount importance [5]. As the primary constituent of the outer membrane, this amphiphilic macromolecule possesses a tripartite architecture: a membrane-anchoring glycolipid (lipid A), a core oligosaccharide, and a highly variable O-polysaccharide chain. Based on their carbohydrate composition, LPSs can be classified as smooth (S-type), containing all three domains, or rough (R-type, lipooligosaccharide, LOS), lacking the O-polysaccharide [6,7]. The structural diversity of LPSs, evident across different genera and even among individual strains, directly reflects the evolutionary strategies employed by bacteria to reinforce their cell envelope. Consequently, deep-sea bacterial LPS molecules frequently display unconventional structural modifications that hold profound interest for evolutionary biochemistry and structural biology [8,9,10]. Beyond their ecological functions, these unique macromolecular variations possess significant biomedical relevance, particularly through their interactions with the mammalian innate immune system. The lipid A moiety serves as the primary ligand for the Toll-like receptor 4/myeloid differentiation factor 2 (TLR4/MD-2) complex [11,12,13]. While LPS from terrestrial pathogens typically triggers a robust, potentially fatal pro-inflammatory cytokine cascade, the immunological outcome of this recognition is strictly dictated by the specific chemical structure of the lipid A. Variations in acylation patterns, degree of phosphorylation, or carbohydrate modifications can alter the biological response across a broad spectrum, ranging from potent agonism to complete signaling silence or active receptor antagonism. Discovering structural variants capable of binding TLR4/MD-2 to block classical agonists offers a strategy for designing targeted immunomodulators to control excessive inflammation [14,15,16,17]. Because deep-sea bacteria are ecologically isolated from terrestrial hosts, their interactions with mammalian immune receptors may differ from those of classical human pathogens, representing a potential source of unique lipid A structural variants. Exploration of these remote marine habitats may facilitate the discovery of novel lipid A chemotypes that could serve as structural templates and provide a molecular basis for the development of immunotherapeutics inspired by extremophilic marine organisms.

Following the recent structural elucidation and immunological characterization of the lipid A from the coastal strain *Rheinheimera japonica* KMM 9513^T^ [18], it is of interest to examine deep-sea representatives of this genus. To expand the current understanding of deep-sea bacterial glycoconjugates, the present investigation targets the LOS produced by *R. pacifica* KMM 1406^T^, a strain isolated from the Northwest Pacific Ocean at a depth of 5000 m [19]. Although our earlier study characterized a cell-surface polysaccharide from this strain as a putative O-antigen [20], implying an S-type LPS, the present structural analyses indicate that this molecule functions as a distinct capsular polysaccharide (CPS), a feature increasingly recognized among deep-sea isolates [21]. Here, we report that the outer membrane of *R. pacifica* KMM 1406^T^ features an R-type LPS (LOS), which coexists with the CPS. To our knowledge, this is the first report of the complete structures of both the lipid A and the core oligosaccharide domains of this LOS.

## 2. Results and Discussion

### 2.1. Isolation and General Characterization of R. pacifica KMM 1406^T^ LOS

Dried cells of *R. pacifica* KMM 1406^T^ were extracted using the phenol–chloroform–petroleum ether (PCP) method [22], followed by enzymatic treatment and ultracentrifugation. Silver-stained SDS-PAGE analysis of the obtained preparation showed a low-molecular-mass smear at the bottom of the gel, characteristic of R-type LPS (LOS) (Figure S1).

Sequentially, the remaining cell debris from the PCP extraction was subjected to isolation of the CPS by hot phenol–water extraction [23]. Subsequent compositional analysis of the CPS demonstrated the absence of LPS, as no detectable levels of canonical LPS markers (3-deoxy-D-*manno*-oct-2-ulosonic acid and 3-hydroxy fatty acids) were found, and silver-stained SDS-PAGE revealed no ladder-like pattern typical of S-type LPS. NMR analysis (Figure S2) of the obtained CPS fraction demonstrated that its structure (Figure S3) was identical to that previously reported [20]. This experimental evidence confirms that the previously described polysaccharide is not covalently linked to the lipid A/core oligosaccharide as an O-antigen, but represents a distinct CPS that remained in the cell debris during the PCP extraction.

In turn, total fatty acid analysis of LOS by GC-MS revealed dodecanoic [12:0], 3-hydroxydodecanoic [12:0(3-OH)], and 3-hydroxytetradecanoic [14:0(3-OH)] acids in a ratio of 1:2.9:4.1 (relative to 12:0, taken as 1). Minor amounts of 3-hydroxyundecanoic [11:0(3-OH)], 3-hydroxytridecanoic [13:0(3-OH)], and 3-hydroxy*iso*tetradecanoic [*i*14:0(3-OH)] acids were also detected in a ratio of 0.2:0.6:0.4 relative to 12:0. To determine the composition of the carbohydrate chain, the LOS was completely deacylated, and the LOS-derived oligosaccharide (OS) fraction was isolated by gel-permeation chromatography. Monosaccharide analysis of the OS by GC-MS of acetylated methyl and (*S*)-2-butyl glycosides indicated the presence of 2-amino-2-deoxy-D-glucose (D-GlcN), D-galactose (D-Gal), and D-glucose (D-Glc). Additionally, 3-deoxy-D-manno-oct-2-ulosonic acid (Kdo) was detected only after treatment of the oligosaccharides with hydrofluoric acid, which indicated that this residue was phosphorylated.

**Figure 1:**
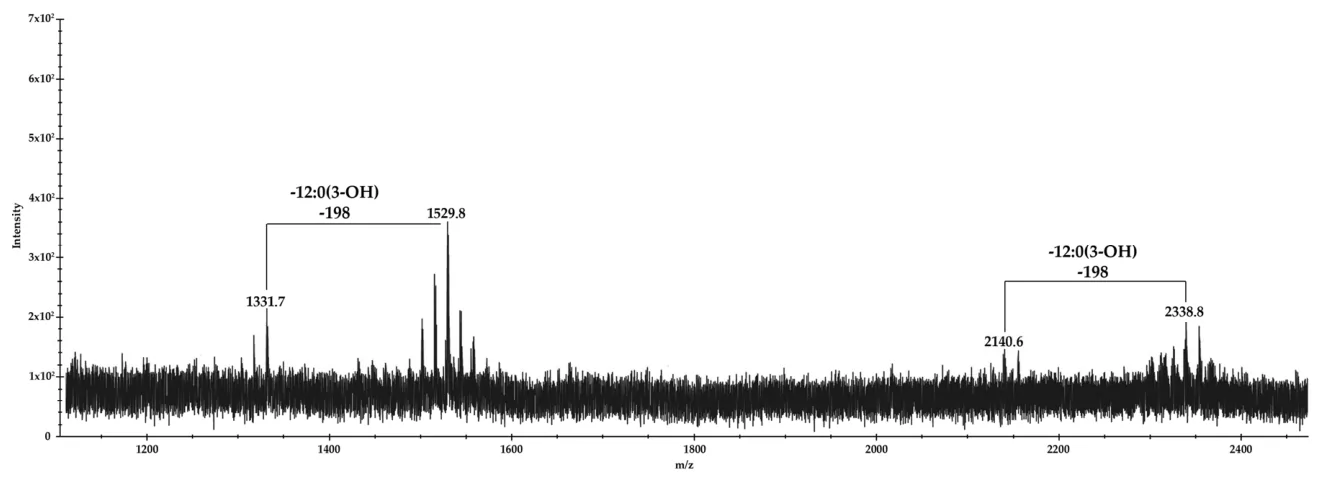
Negative-ion MALDI-TOF mass spectrum of the intact LOS from *R. pacifica* KMM 1406^T^. Mass labels indicate the peaks corresponding to the bis-phosphorylated tetra-acylated (*m/z* 1331.7) and penta-acylated (*m/z* 1529.8) lipid A species, as well as the intact tetra-acylated (*m/z* 2140.6) and penta-acylated (*m/z* 2338.8) LOS molecular ions.

The intact LOS was analyzed by negative-ion matrix-assisted laser desorption/ionization time-of-flight mass spectrometry (MALDI-TOF MS) (Figure 1).

The spectrum contained ion clusters corresponding to both the LOS and lipid A molecules. In the high-molecular-mass region, the peak at *m/z* 2338.8 was attributed to a penta-acylated LOS molecule as a monosodium salt with the composition Gal_2_GlcKdoPGlcN_2_P_2_[14:0(3-OH)]_2_[12:0(3-OH)]_2_(12:0)·Na (calculated [M + Na–2H]^−^ = 2338.1 Da). The peak at *m/z* 2140.6 was assigned to a tetra-acylated isoform lacking one 12:0(3-OH) residue (calculated [M + Na–2H]^−^ = 2140.0 Da). In the lower-mass region, signals from the lipid A domain were observed. The ions at *m/z* 1529.8 and 1331.7 (calculated [M – H]^−^ = 1530.0 and 1331.8 Da, respectively) were assigned to bis-phosphorylated penta- and tetra-acylated lipid A molecules differing by one 12:0(3-OH) unit (Figure 1). It should be noted that although both tetra- and penta-acylated forms were detected in the intact LOS spectrum (Figure 1), the observed peak ratios may not accurately represent their actual physiological abundance in the native bacterial membrane because of possible ionization bias or minor structural degradation during the extraction process.

To determine the structure of the core oligosaccharide domain attached to the lipid A backbone, the completely deacylated OS fraction was studied by NMR spectroscopy.

### 2.2. Structural Elucidation of the Core Oligosaccharide

**Figure 2:**
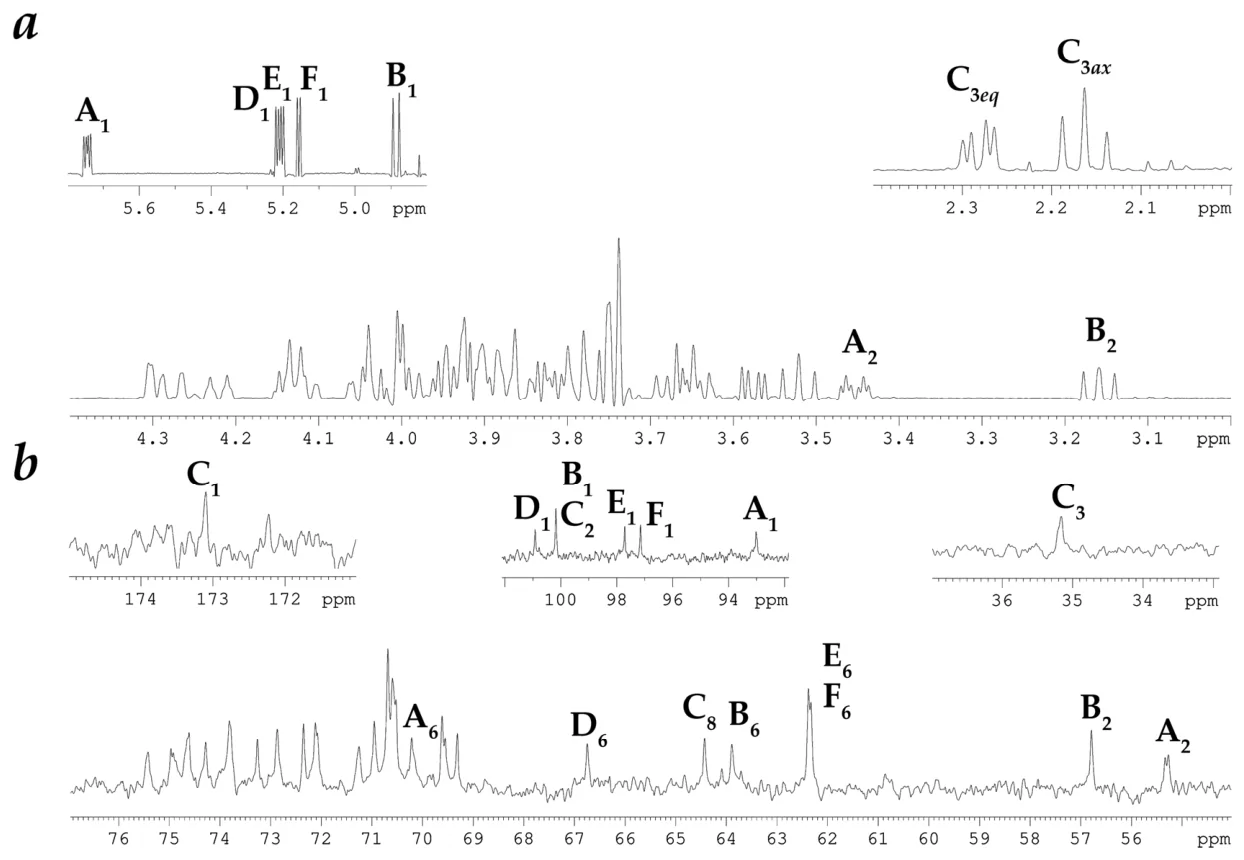
^1^H (**a**) and ^13^C NMR (**b**) spectra of the OS from *R. pacifica* KMM 1406^T^. Numerals refer to atoms in sugar residues, which are denoted by capital letters as outlined in Table 1.

Analysis of the ^1^H NMR spectrum of the OS (Figure 2a, Table 1) revealed five distinct signals within the anomeric region at δ_H_ 5.74 (^3^*J*_H1-H2_ 3.5 Hz, ^3^*J*_H1-P_ 6.2 Hz, H-1 of residue **A**), 5.21 (^3^*J*_H1–H2_ 3.7 Hz, H-1 of residue **D**), 5.20 (^3^*J*_H1–H2_ 3.7 Hz, H-1 of residue **E**), 5.15 (^3^*J*_H1–H2_ 4.0 Hz, H-1 of residue **F**) and 4.88 (^3^*J*_H1–H2_ 8.5 Hz, H-1 of residue **B**). Furthermore, characteristic methylene signals appearing in the higher field at δ_H_ 2.28 (^3^*J*_H3eq-H4_ 4.7 Hz, H-3*eq* of residue **C**) and 2.16 (^3^*J*_H3ax-H4_ 12.4 Hz, H-3*ax* of residue **C**) confirmed the presence of a 3-deoxy-D-*manno*-oct-2-ulosonic acid (Kdo) unit. The relatively small vicinal coupling constants calculated for residues **A**, **D**, **E**, and **F** point to their α-anomeric configurations, whereas the larger splitting observed for residue **B** is indicative of a β-linked monosaccharide. The α-configuration of the Kdo*p* residue was established based on the difference (0.12 ppm) between H-3*eq* and H-3*ax* chemical shifts [24].

In the ^13^C NMR spectrum of the OS (Figure 2b, Table 1), six resonances were observed within the anomeric region. These included the ketosidic C-2 carbon of the Kdo*p* residue (**C**) at δ_C_ 100.1, as well as five other anomeric signals at δ_C_ 100.8 (C-1 of residue **D**), 100.1 (C-1 of residue **B**), 97.6 (C-1 of residue **F**), 97.1 (C-1 of residue **E**), and 92.9 (C-1 of residue **A**). Two signals characteristic of nitrogen-bearing carbons appeared at δ_C_ 56.7 and 55.2 (C-2 of residues **B** and **A**, respectively). Data from the DEPT-135 experiment facilitated the assignment of methylene and hydroxymethyl groups, which showed inverted phases. Among these, six signals were attributed to hydroxymethyl carbons at δ_C_ 70.2 (C-6 of residue **A**), δ_C_ 66.7 (C-6 of residue **D**), δ_C_ 64.4 (C-8 of residue **C**), δ_C_ 63.8 (C-6 of residue **B**), δ_C_ 62.3 (C-6 of residue **E**), and δ_C_ 62.3 (C-6 of residue **F**). The single high-field methylene resonance at δ_C_ 35.1 was assigned to C-3 of the Kdo unit (**C**). Additionally, the ^31^P NMR spectrum () demonstrated three phosphate ester groups, appearing as two peaks at δ_P_ −1.57 and 0.31, with the latter displaying a double integral intensity.

**Figure 3:**
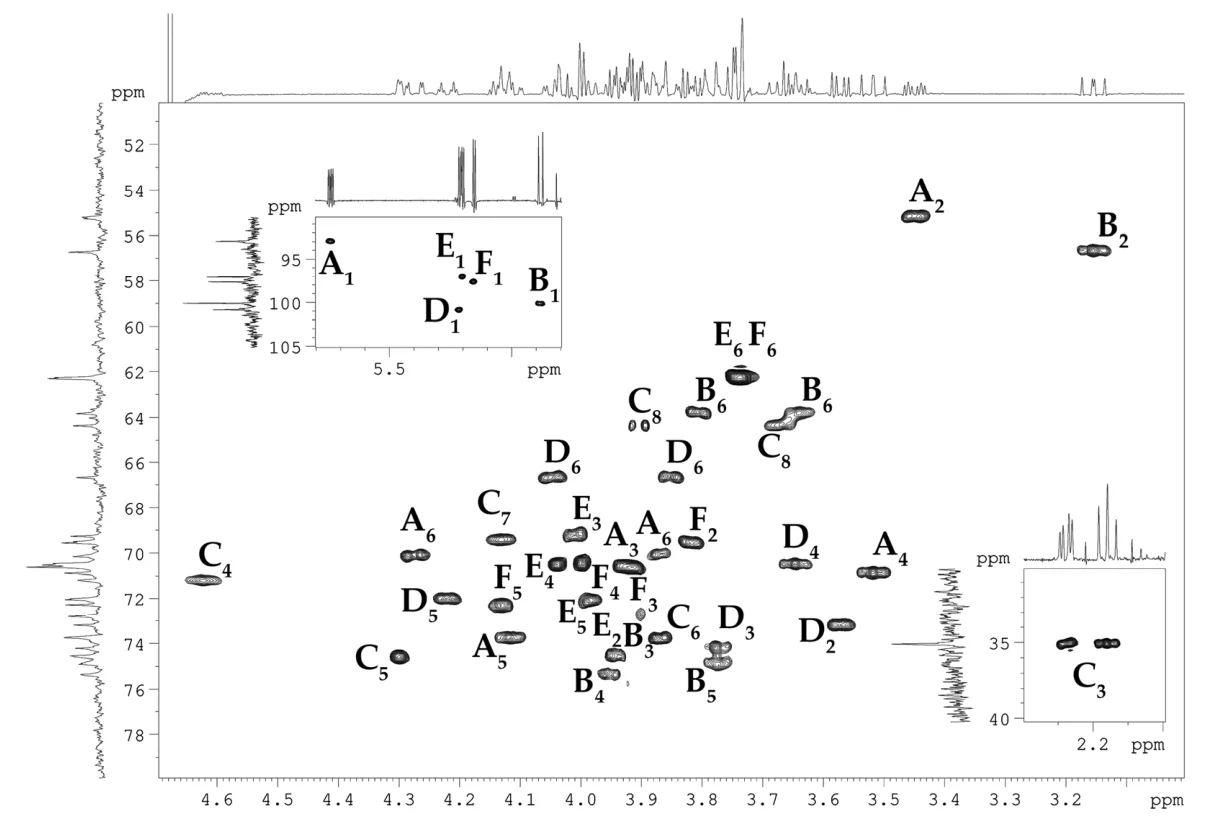
^1^H,^13^C HSQC spectrum of the OS from *R. pacifica* KMM 1406^T^. Numerals refer to atoms in sugar residues, which are denoted by capital letters as outlined in Table 1.

**Figure 4:**
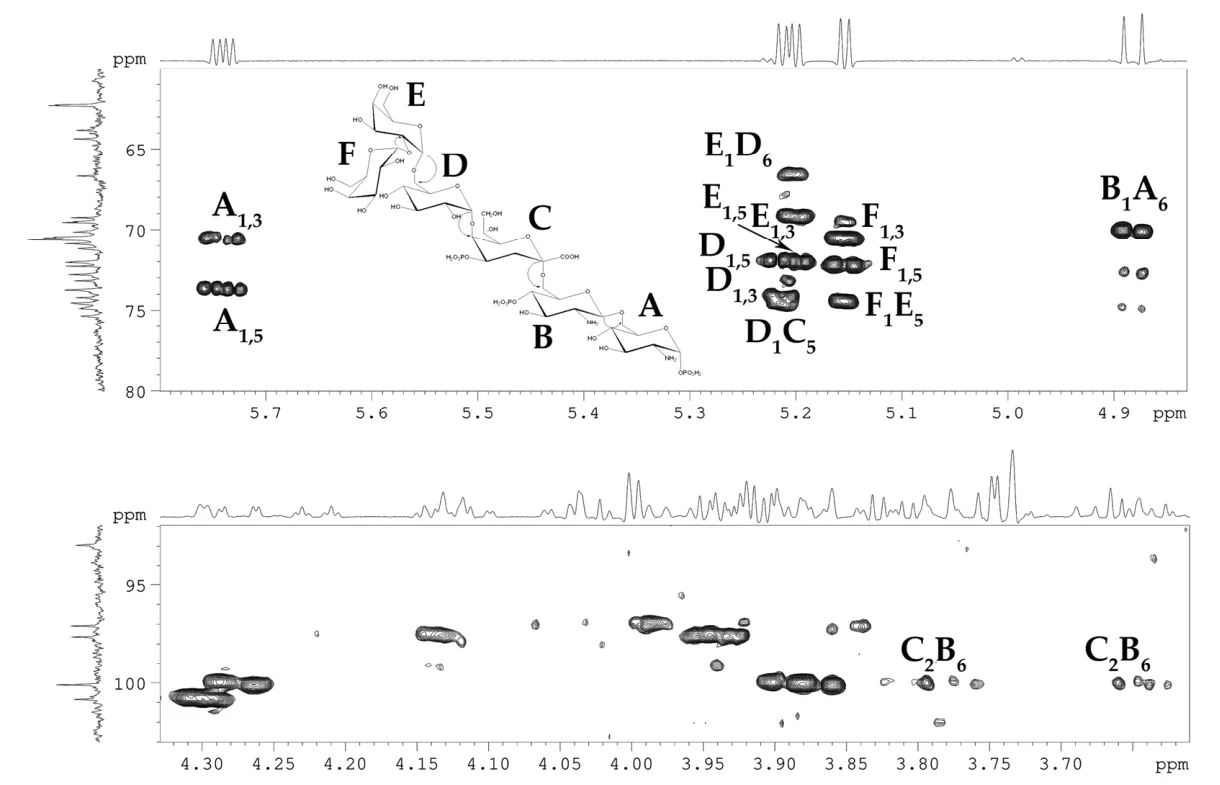
Fragment of ^1^H,^13^C HMBC spectrum of the OS from *R. pacifica* KMM 1406^T^. Numerals refer to atoms in sugar residues, which are denoted by capital letters as outlined in Table 1. Key inter-residue HMBC correlations are indicated by arrows.

Comprehensive structural elucidation of the OS (Table 1) was performed using a series of two-dimensional NMR experiments. Individual spin systems were identified through the analysis of homonuclear ^1^H,^1^H COSY and ^1^H,^1^H TOCSY spectra, whereas the corresponding carbon resonances were determined via heteronuclear ^1^H,^13^C HSQC correlations (Figure 3). The sequence of the monosaccharide residues and their linkage positions were established using ^1^H,^13^C HMBC (Figure 4) and ^1^H,^1^H ROESY experiments, ensuring the unambiguous assignment of all proton and carbon atoms within the OS. Furthermore, the exact positions of the phosphate groups were determined using a heteronuclear ^1^H,^31^P HMBC experiment and were independently verified using heteronuclear carbon-phosphorus coupling constants (^2^*J*_C-P_, Table 1).

Specifically, residues **A** and **B** were assigned to the two D-Glc*p*N units constituting the disaccharide backbone of the lipid A moiety. This assignment was based on their characteristic H-2/C-2 cross-peaks at δ_H_/δ_C_ 3.45/55.2 and 3.15/56.7 in the ^1^H,^13^C HSQC spectrum (Figure 3), respectively. Phosphorylation of both D-Glc*p*N residues was demonstrated by heteronuclear ^1^H,^31^P HMBC correlations connecting H-1 of residue **A** and H-4 of residue **B** to their respective phosphate groups at δ_H_/δ_P_ 5.74/–1.57 and 3.95/0.31, respectively. The β-(1→6) linkage of these amino sugars was confirmed by inter-residue NOE correlations connecting H-1 of residue **B** with the H-6 protons of residue **A** at δ_H_/δ_H_ 4.88/4.27, 3.87. This connectivity was independently validated by a long-range scalar correlation from H-1 of residue **B** to C-6 of residue **A** at δ_H_/δ_C_ 4.88/70.2 in the ^1^H,^13^C HMBC spectrum (Figure 4). Residue **B** was found to be substituted at the *O*-6 position by the Kdo*p* residue (**C**). This substitution was supported by a weak downfield shift of the C-6 signal of residue **B**, characteristic of ketose glycosylation and the HMBC correlation between C-2 of residue **C** and H-6 of residue **B** at δ_C_/δ_H_ 100.1/3.81, 3.64.

Residue **C** was identified as an α-Kdo*p* unit, with the assignment initiated from the characteristic high-field methylene protons at δ_H_ 2.28 and 2.16. In the ^1^H,^1^H TOCSY spectrum, these H-3 signals correlated with protons at δ_H_ 4.62 and 4.30, which were sequentially assigned to H-4 and H-5, respectively. The glycosylation site of the α-Kdo*p* residue was determined to be at the *O*-5 position, as deduced from the significant downfield shift of its C-5 resonance to δ_C_ 74.5 relative to the unsubstituted reference monosaccharide [24]. This site was found to be substituted by residue **D**, as corroborated by the corresponding inter-residue NOE contact at δ_H_/δ_H_ 5.21/4.30, along with the HMBC correlation at δ_H_/δ_C_ 5.21/74.5. Furthermore, the downfield positions of the H-4 (δ_H_ 4.62) and C-4 (δ_C_ 71.2) resonances strongly suggested the presence of a phosphate group at this position. This phosphorylation pattern was unequivocally confirmed by the ^1^H,^31^P HMBC spectrum, which revealed a clear cross-peak between H-4 and a phosphate group at δ_H_/δ_P_ 4.62/0.31.

Residue **D** was identified as an α-D-Glc*p* unit. Analysis of the ^1^H,^1^H TOCSY spectrum revealed a spin system characteristic of the *gluco*-configuration. Based on a comprehensive analysis of the homonuclear and heteronuclear NMR data, complete assignment of the proton and carbon chemical shifts for this residue was achieved (Table 1). The significant downfield shift of the C-6 resonance to δ_C_ 66.7, compared to the corresponding value for an unsubstituted monosaccharide [25], indicated that residue **D** was glycosylated at the *O*-6 position. This linkage site was unambiguously verified by inter-residue cross-peaks in the ^1^H,^1^H ROESY and ^1^H,^13^C HMBC (Figure 4) spectra at δ_H_/δ_H_ 5.20/3.86, 4.05 and δ_H_/δ_C_ 5.20/66.7, respectively, which correlated H-1 of residue **E** with H-6 and C-6 of residue **D**.

Finally, residues **E** and **F** were assigned to a 2-substituted and a terminal α-D-Gal*p* unit, respectively. The *galacto*-configuration of these residues was deduced from the intrinsic *J*-coupling limitations in the ^1^H,^1^H TOCSY spectrum, which showed characteristic magnetization transfer from H-1 only up to H-4. The C-2 resonance of residue **E** shifted downfield to δ_C_ 74.6 compared to that of an unsubstituted galactose [25], demonstrating glycosylation at the *O*-2 position. This substitution pattern was further confirmed by inter-residue ^1^H,^1^H ROESY and ^1^H,^13^C HMBC (Figure 4) correlations at δ_H_/δ_H_ 5.15/3.94 and δ_H_/δ_C_ 5.15/74.6, respectively, which correlated H-1 of residue **F** with H-2 and C-2 of residue **E**. Analysis of the NMR data for residue **F** revealed chemical shifts close to those of the unsubstituted monosaccharide [25], thereby confirming its identity as a terminal unit.

Based on the collective spectroscopic evidence, the complete structure of the core OS from *R. pacifica* KMM 1406^T^ was established as follows:

α-D-Gal*p*-(1→2)-α-D-Gal*p*-(1→6)-α-D-Glc*p*-(1→5)-α-Kdo*p*4P-(2→6)-β-D-Glc*p*N4P-(1→6)-α-D-Glc*p*N1P

To determine the chemical structure of the lipid A moiety and establish the acyl chain substitution pattern, the isolated LOS was subjected to mild acid hydrolysis. The glycolipid fraction was subsequently investigated by MALDI-TOF MS and MS/MS.

### 2.3. Structural Elucidation of the Lipid A

**Figure 5:**
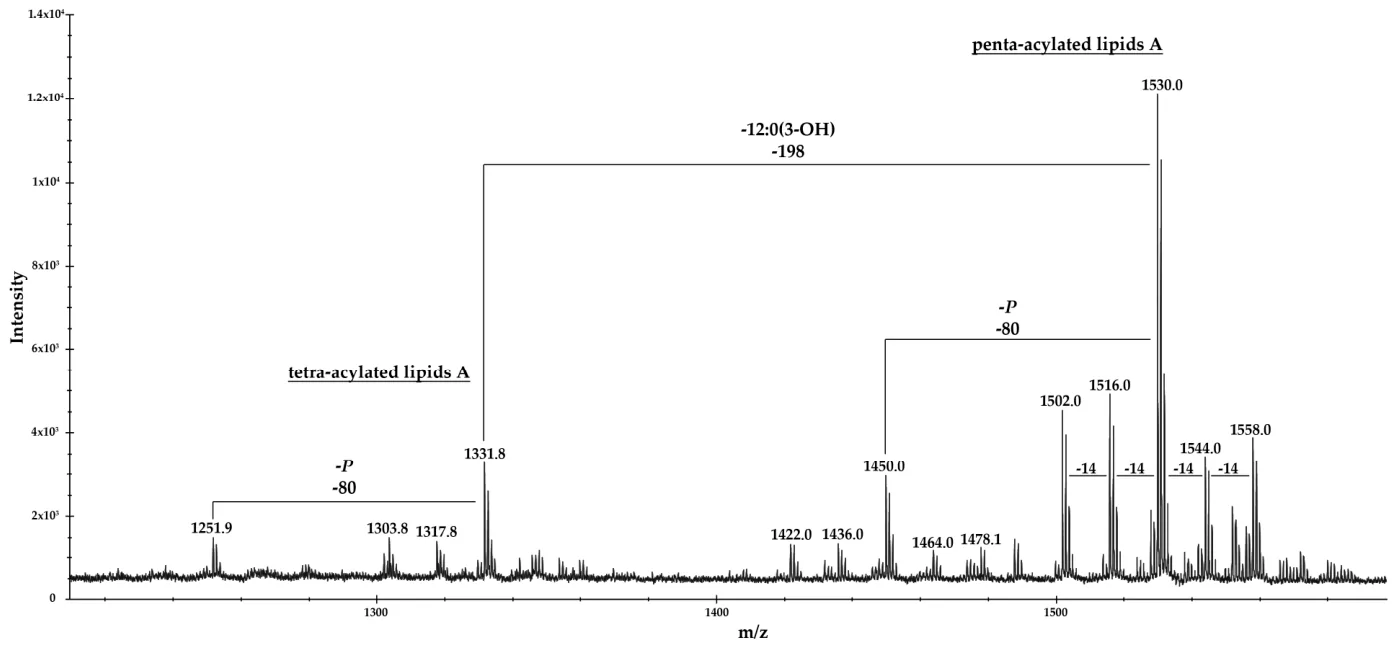
Negative-ion MALDI-TOF mass spectrum of the lipid A from *R. pacifica* KMM 1406^T^ recorded in reflector mode.

Analysis of the negative-ion MALDI-TOF mass spectrum of the lipid A revealed four peak clusters, resulting from structural variations in fatty acid composition and partial dephosphorylation during mild acid hydrolysis (Figure 5). The deprotonated molecular ions [M − H]^−^ at *m/z* 1530.0, 1450.0, 1331.8, and 1251.9 were assigned to bis- and mono-phosphorylated variants of penta- and tetra-acylated lipid A species, respectively (Table 2). Specifically, the peak at *m/z* 1530.0 was attributed to a bis-phosphorylated, penta-acylated lipid A molecular species containing two 14:0(3-OH), two 12:0(3-OH), and one 12:0 fatty acid unit, whereas the corresponding mono-phosphorylated ion was observed at *m/z* 1450.0. Furthermore, the tetra-acylated glycoform region exhibited a bis-phosphorylated peak at *m/z* 1331.8, which lacks one 12:0(3-OH) acyl chain compared to the penta-acylated forms, along with its mono-phosphorylated counterpart at *m/z* 1251.9. Additionally, these principal ions were accompanied by satellite peaks differing by ±14 Da. While these shifts are consistent with general acyl-chain microheterogeneity (differing by a methylene group, –CH_2_–), the individual minor species cannot be unambiguously identified, as their low abundance precluded individual high-resolution tandem MS/MS validation. Moreover, the quantitative ratios of these lipid A variants should be interpreted with caution, as the mild acid hydrolysis used to release the glycolipid fraction can cause partial loss of labile ester-linked acyl chains or selective degradation of specific glycoforms, potentially leading to underestimation of more labile species.

**Figure 6:**
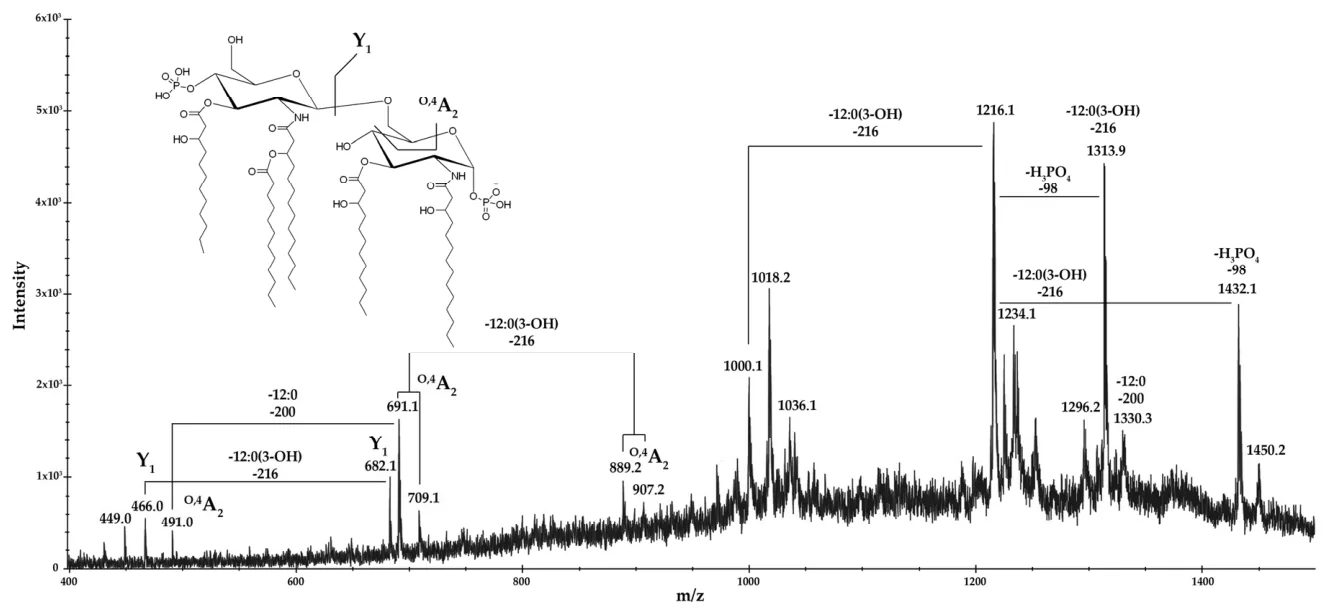
MALDI-TOF tandem mass spectrometry (MS/MS) analysis of the penta-acylated lipid A species at *m/z* 1530.0. Fragment assignments are indicated.

MS/MS analysis in the negative-ion mode was employed to establish the distribution of the acyl chains on the glucosamine disaccharide backbone of the lipid A. Fragments involving both glycosidic cleavage and intra-ring bonds were designated according to the nomenclature established by Costello and Vath [26]. In particular, the MS/MS spectrum of the precursor molecular ion at *m/z* 1530.0 (Figure 6) featured a fragment at *m/z* 1432.1, indicating the elimination of a phosphate group. Losses of the 12:0 and 12:0(3-OH) fatty acyl chains from the parent ion yielded fragment peaks at *m/z* 1330.3 and 1313.9, respectively. The signal at *m/z* 1216.1 was identified as a product ion arising from the concurrent loss of both a phosphate moiety and a 12:0(3-OH) acyl chain. Notably, the spectrum lacked any fragment peaks associated with the release of an *O*-acyloxyacyl group, demonstrating that the secondary 12:0 fatty acid was attached directly to the amide-linked acyl chain. The Y_1_ fragment ion observed at *m/z* 682.1, generated by the cleavage of the glycosidic linkage within the disaccharide backbone, indicated that the proximal D-Glc*p*N unit was di-acylated with 14:0(3-OH) and 12:0(3-OH) fatty acids. Consequently, the distal D-Glc*p*N unit carried three acyl chains, identified as 14:0(3-OH), 12:0(3-OH), and 12:0. In agreement with this distribution, the fragment peak at *m/z* 466.0 was assigned to the Y_1_ ion after the additional loss of the 12:0(3-OH) fatty acid chain, further confirming the acylation pattern of the proximal D-Glc*p*N. The proposed acylation pattern of the distal D-Glc*p*N was independently supported by a series of cross-ring fragmentation products, including characteristic ^O,4^A_2_ ions at *m/z* 907.2 and 889.2. Subsequent elimination of the 12:0(3-OH) acyl chain from these cross-ring fragments yielded diagnostic peaks at *m/z* 709.1 and 691.1, which underwent further fragmentation to produce the ion at *m/z* 491.1, thereby unequivocally confirming the acylation layout of the non-reducing D-Glc*p*N.

**Figure 7:**
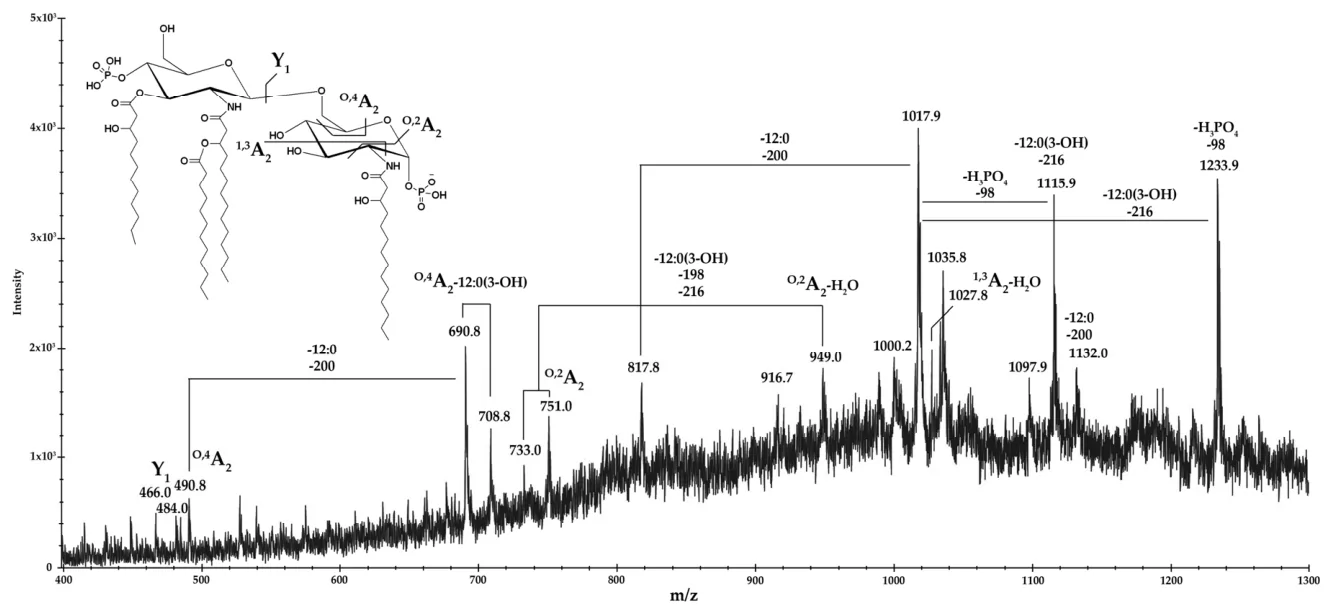
MALDI-TOF tandem mass spectrometry (MS/MS) analysis of the tetra-acylated lipid A species at *m/z* 1331.8. Fragment assignments are indicated.

The negative-ion MS/MS spectrum (Figure 7) of the precursor ion at *m/z* 1331.8, corresponding to the bis-phosphorylated tetra-acylated lipid A species, provided fragment peaks that determined its chemical structure. This species contains two 14:0(3-OH) acyl chains, one 12:0(3-OH), and one 12:0 unit. The elimination of a phosphate group, a 12:0, and a 12:0(3-OH) fatty acid chain from the precursor ion yielded peaks at *m/z* 1233.9, 1132.0, and 1115.9, respectively. The peak observed at *m/z* 1017.9 was assigned to a fragment resulting from the simultaneous loss of both a phosphate group and a 12:0(3-OH) chain. Further structural analysis was achieved by the detection of Y_1_ and ^O,4^A_2_ fragment ions at *m/z* 484.0 and 708.8 [corresponding to a loss of 198 Da, 12:0(3-OH)], respectively. These ions demonstrated that the proximal D-Glc*p*N unit is mono-acylated with a single 14:0(3-OH) chain, while the distal D-Glc*p*N unit retains the identical tri-acylation pattern found in the penta-acylated lipid A variant. Crucially, the appearance of the ^1,3^A_2_ and ^O,2^A_2_ cross-ring fragment ions at *m/z* 1027.8 and 949.0, respectively, confirmed that the *O*-3 hydroxyl position of the reducing D-Glc*p*N unit is unsubstituted [27]. These diagnostic fragmentation pathways verified the specific distribution of the acyl chains on the disaccharide backbone.

The MS/MS spectra of the mono-phosphorylated precursor ions at *m/z* 1450.0 and 1251.9 demonstrated both the sequential elimination of the acyl substituents and the presence of the corresponding cross-ring fragments. The absence of characteristic Y_1_ ions in the MS/MS spectra indicated the loss of the phosphate group from the *O*-1 position of the proximal D-Glc*p*N residue, which was cleaved during mild acid hydrolysis.

Finally, to confirm the proposed acylation pattern, an aliquot of the isolated LOS was subjected to mild alkaline hydrolysis with dilute ammonium hydroxide to selectively remove the primary *O*-linked acyl chains [28]. As anticipated, the resulting MALDI-TOF mass spectrum revealed new peak clusters corresponding to bis- and mono-phosphorylated tri-acylated lipid A species at *m/z* 1133.6 and 1053.6. These lipid A variants contained two 14:0(3-OH) and one 12:0 fatty acid units, confirming their identity as the amide-linked acyl chains.

**Figure 8:**
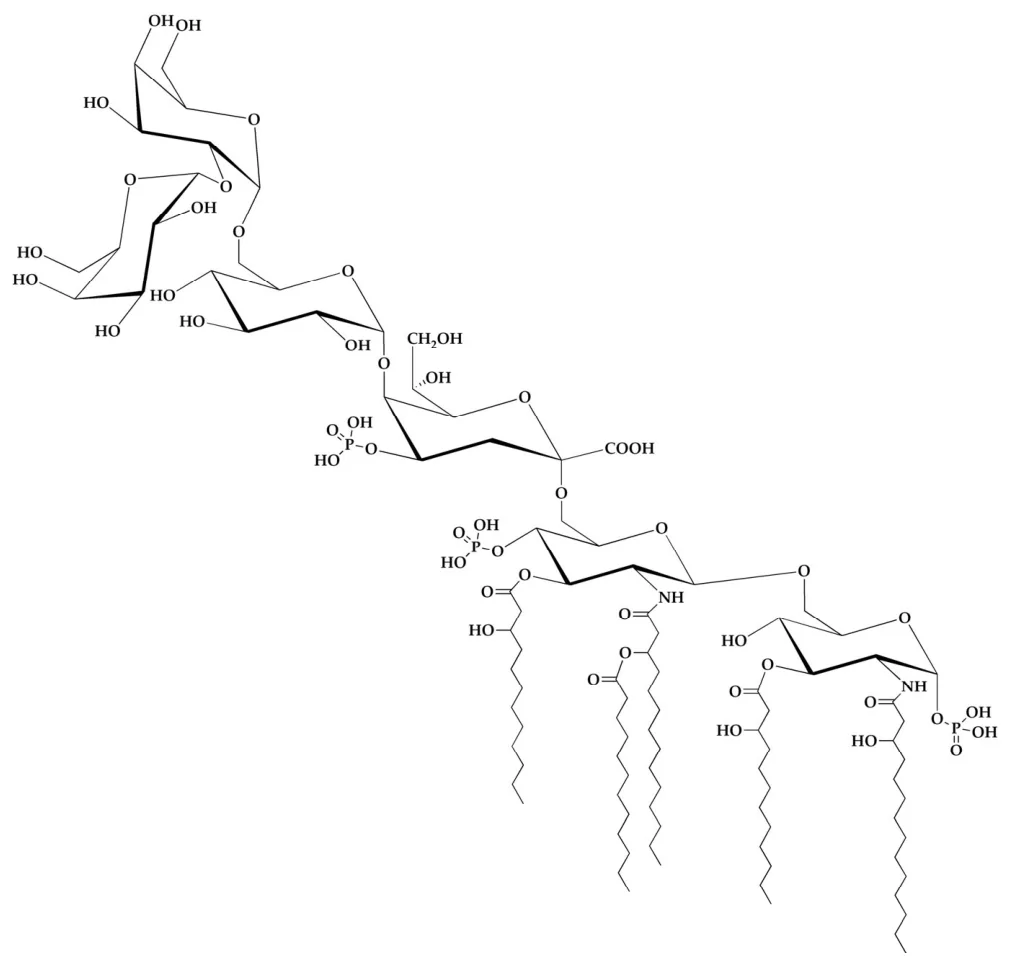
Proposed structure of the LOS from *R. pacifica* KMM 1406^T^.

Consequently, a combined analysis of the compositional data, NMR spectroscopy, and MALDI-TOF mass spectrometry enabled the structural determination of the LOS from *R. pacifica* KMM 1406^T^. The established structure, with the predominant lipid A species, is schematically presented in Figure 8.

## 3. Conclusions

Structural variations in the LPSs of Gram-negative bacteria frequently reflect environmental adaptations required to maintain cell envelope stability under extreme abiotic conditions [5]. Chemically, these adaptations typically involve a lower degree of lipid A acylation and phosphorylation, and the presence of branched, unsaturated, or shortened fatty acids [5,8,9,10,18,27,29,30,31,32,33,34,35,36,37,38,39,40,41,42]. Furthermore, in marine and other extremophilic Gram-negative bacteria, core oligosaccharides typically display short carbohydrate chains modified with negatively charged functional groups [5,10,34,36,43,44,45,46].

In the present study, we established for the first time the complete structure of the LOS of *R. pacifica* KMM 1406^T^, isolated at a depth of 5000 m. From a structural perspective, the LOS of *R. pacifica* KMM 1406^T^ is characterized by a conserved chemical composition. The hydrophobic lipid A domain consists primarily of saturated, straight-chain fatty acids, and only a minor amount of *i*14:0(3-OH) acid was detected. Furthermore, although the core OS features a truncated carbohydrate chain, the presence of a phosphorylated Kdo residue represents a common structural constituent rather than a unique evolutionary modification.

Nevertheless, the determined structure enriches the database of LOSs from marine extremophiles. Notably, the combination of a low acylation degree and a short core oligosaccharide, despite the predominance of saturated straight-chain fatty acids, suggests a potential mechanism for modulating membrane fluidity through reduced acyl chain packing density rather than through incorporation of branched or unsaturated chains, as is observed in some other psychrophiles or deep-sea isolates [8,9,10,32,33,35,42,45]. Although this architecture may contribute to cell envelope stability in abyssal environments, this hypothesis requires experimental validation under simulated deep-sea conditions.

Interestingly, comparison with the recently characterized lipid A from the phylogenetically related coastal strain *R. japonica* KMM 9513^T^ [18] reveals striking structural differences that may reflect distinct ecological strategies. While *R. japonica* KMM 9513^T^ lipid A exhibits pronounced microheterogeneity, including branched (iso/anteiso) and unsaturated (14:1(3-OH)) acyl chains, isobaric species, and variability in acyl chain length, the deep-sea isolate *R. pacifica* KMM 1406^T^ displays a remarkably simplified lipid A structure with saturated straight-chain fatty acids. This compositional economy, combined with a shorter carbohydrate core and a lower overall acylation degree, may represent an adaptive strategy to conserve biosynthetic resources under the nutrient-limited conditions of the deep sea, while maintaining sufficient membrane fluidity through reduced acyl chain packing density. However, caution is warranted regarding the quantitative interpretation of the lipid A acylation profile. The established proportions of tetra- and penta-acylated species represent the state of the isolated macromolecule and may be partially influenced by procedural factors, such as chemical treatments during isolation and derivatization. Moreover, since the bacterium was cultivated under standard laboratory conditions, the described LOS structure represents a basal laboratory phenotype and cannot be definitively designated as the fully adaptive deep-sea state. The proposed relationship between the LOS structural features and deep-sea survival must be treated strictly as a hypothesis. Further cultivation experiments under simulated deep-sea conditions are mandatory to clarify the true metabolic plasticity of the cell envelope in *R. pacifica* KMM 1406^T^.

From a functional perspective, while the *R. japonica* KMM 9513^T^ LPS was shown to act as a TLR4 antagonist with immunomodulatory properties [18], the immunological activity of the *R. pacifica* KMM 1406^T^ LOS remains to be explored, opening intriguing perspectives for comparative structure–activity studies within the genus *Rheinheimera*.

## 4. Materials and Methods

### 4.1. Bacterial Strain, Isolation and Purification of the LOS, OS, and Lipid A

The strain *R. pacifica* KMM 1406^T^ was cultivated following previously described parameters [47]. Extraction of 7.4 g of dried bacterial cells by the PCP method [22] afforded 88.7 mg of crude LOS. The remaining bacterial cells were subjected to hot phenol–water extraction [23] to yield crude CPS (416 mg). The preparation of LOS was analyzed by SDS-PAGE [48] using a 20% polyacrylamide gel, followed by staining with silver nitrate [49]. For further analysis, a 35 mg aliquot of the LOS sample was fully de-O- and de-N-acylated as described previously [10,43], yielding 5.2 mg of OS. To prepare the lipid A sample [50], a 20 mg aliquot of LOS was hydrolyzed in an acetate buffer (pH 4.4) at 100 °C for 3 h, followed by extraction with a Bligh–Dyer mixture (chloroform/methanol/water, 2:2:1.8, *v*/*v*/*v*). The chloroform phase was evaporated to dryness, yielding 3.0 mg of lipid A for further analysis.

### 4.2. Chemical Analysis of the LOS

Monosaccharide composition was determined by converting the sugars in the OS sample into peracetylated *O*-methyl glycosides as described elsewhere [43]. Methanolysis followed by extraction with *n*-hexane and acetylation of the methanolic phase was performed. The organic phase obtained from the hexane extraction contained fatty acids derivatized as FAMEs, which were directly analyzed by GC-MS. The relative molar ratios of fatty acids were calculated from the peak areas of their methyl esters, normalized to the dodecanoic acid (12:0) peak. The absolute configuration of the monosaccharides was inferred by analyzing their acetylated (*S*)-2-butyl glycosides [51]. All derivatives were analyzed using a Hewlett-Packard 5890 chromatograph (Conquer Scientific, Poway, CA, USA) coupled with a Hewlett-Packard 5973 mass spectrometer (USA) equipped with an HP-5MS capillary column under the temperature programs described previously [43]. To obtain the dephosphorylated OS, the sample (1 mg) was treated with aqueous 48% HF (0.1 mL, 4 °C, 16 h); the HF was then evaporated, and the modified OS was analyzed as described above.

### 4.3. NMR Spectroscopy and MALDI-TOF MS Analysis

The OS and CPS were analyzed by ^1^H and ^13^C NMR spectroscopy using a Bruker Avance-III spectrometer (700.13 MHz for ^1^H, 176.04 MHz for ^13^C; Bruker, Karlsruhe, Germany). Spectra were acquired at 30 °C in 99.95% D_2_O, with acetone (δ_C_ 31.45, δ_H_ 2.225) as an internal reference standard. The ^1^H,^1^H-TOCSY and ^1^H,^1^H-ROESY spectra were recorded with a 180 ms duration of MLEV-17 spin-lock and a 300 ms mixing time, respectively. ^1^H,^13^C-HMBC and ^1^H,^31^P-HMBC were optimized for an 8 Hz long-range constant. MALDI-TOF mass spectra were acquired on an ULTRAFLEX III (Bruker, Bremen, Germany) mass spectrometer with a solid-state laser (SmartBeam,355 nm), reflector, and potential LIFT (laser-induced forward transition) modes of operation. All spectra were recorded in the negative-ion reflector mode using an acceleration voltage of 20 kV and a reflector voltage of 22 kV. For intact LOS and lipid A profiling, data were accumulated from 1000 laser shots per spectrum at a laser power adjusted slightly above the threshold. Mass calibration was performed externally using a characterized lipid A [10,43,50] in the mass range of interest, yielding a mass accuracy of ±0.2 Da. Tandem mass spectrometry (MS/MS) experiments were conducted under LIFT (laser-induced forward transition) acceleration parameters. The precursor ion selection window was set to ±7 Da, and the fragment ions were accelerated via a two-stage LIFT cell operating at 8.0 kV and 19.0 kV, respectively, to ensure the maximum mass resolution of product ions. A solution of trihydroxyacetophenone (THAP) in CH_3_OH/aq 0.1% trifluoroacetic acid (TFA)/CH_3_CN (7:2:1, *v*/*v*/*v*) at a concentration of 75 mg/mL was used as the matrix. The lipid A sample was dissolved in chloroform/methanol (3:1, *v*/*v*). The LOS sample was dissolved in 2-propanol/water 1:1 (*v*/*v*).

## Figures and Tables

**Table 1:** ^1^H and ^13^C chemical shifts of the OS from *R. pacifica* KMM 1406^T^, (δ, ppm).

Sugar Residue	H-1 C-1	H-2 C-2	H-3eq,ax C-3	H-4 C-4	H-5 C-5	H-6a,b C-6	H-7a,b C-7	H-8a,b C-8
→6)-α-D-Glc*p*N1P **A**	5.74 ^3^*J*_H1-H2_ 3.5 Hz ^3^*J*_H1-P_ 6.2 Hz 92.9 ^2^*J*_C1-P_ 5.7 Hz	3.45 55.2	3.92 70.6	3.52 70.9	4.13 73.7	4.27, 3.87 70.2		
→6)-β-D-Glc*p*N4P-(1→ **B**	4.88 ^3^*J*_H1–H2_ 8.5 Hz 100.1	3.15 56.7	3.91 72.8	3.95 75.4 ^2^*J*_C4-P_ 5.9 Hz	3.78 74.9	3.81, 3.64 63.8		
→5)-α-Kdo*p*4P-(1→ **C**	173.1	100.1	2.28, 2.16 ^3^*J*_H3ax-H4_ 12.4 Hz ^3^*J*_H3eq-H4_ 4.7 Hz 35.1	4.62 71.2 ^2^*J*_C4-P_ 6.1 Hz	4.30 74.5	3.87 73.8	4.13 69.5	3.91, 3.66 64.4
→6)-α-D-Glc*p*-(1→ **D**	5.21 ^3^*J*_H1–H2_ 3.7 Hz 100.8	3.57 73.2	3.78 74.2	3.65 70.5	4.22 72.0	4.05, 3.86 66.7		
→2)-α-D-Gal*p*-(1→ **E**	5.20 ^3^*J*_H1–H2_ 3.7 97.1	3.94 74.6	4.02 69.2	4.04 70.5	3.98 72.1	3.74, 3.74 62.3		
α-D-Gal*p*-(1→ **F**	5.15 ^3^*J*_H1–H2_ 4.0 Hz 97.6	3.82 69.5	3.92 70.6	4.00 70.5	4.13 72.3	3.74, 3.74 62.3		

**Table 2:** The main ion peaks observed in the MALDI-TOF MS spectrum reported in Figure 5, the predicted masses, and the proposed interpretation of the substituting fatty acids, and phosphate (P), on the lipid A backbone. The observed masses listed in the table are compared to the calculated monoisotopic mass (predicted mass, Da) of each ion based on the proposed lipid A structures.

Lipid A (Figure 6)			
Calculated Neutral Mass (*M*, Da)	Calculated [M − H]^−^ (*m/z*)	Observed Ion Peaks (*m/z*)	Proposed Composition
1531.0	1530.0	1530.0	GlcN_2_, P_2_, [14:0(3-OH)]_2_, [12:0(3-OH)]_2_, 12:0
1451.0	1450.0	1450.0	GlcN_2_, P, [14:0(3-OH)]_2_, [12:0(3-OH)]_2_, 12:0
1332.8	1331.8	1331.8	GlcN_2_, P_2_, [14:0(3-OH)]_2_, 12:0(3-OH), 12:0
1252.8	1251.8	1251.9	GlcN_2_, P, [14:0(3-OH)]_2_, 12:0(3-OH), 12:0

## Data Availability

Dataset available on request from the authors.

## Supplementary Materials

The following supporting information can be downloaded at: https://www.mdpi.com/article/10.3390/md24090322/s1, Figure S1. Silver-stained electrophoregram: lane 1 - *E. coli* 0111:B4 LPS 5 μg, lane 2 – *R. pacifica* KMM 1406^T^ 8 μg, lane 3 – *R. pacifica* KMM 1406^T^ 4 μg; Figure S2. ^1^H (**A**) and ^13^C NMR (**B**) spectra of the CPS from *R. pacifica* KMM 1406^T^; Figure S3. The structure of the CPS from *R. pacifica* KMM 1406^T^.

## References

[1] Kallmeyer J., Pockalny R., Adhikari R.R., Smith D.C., D’Hondt S. (2012). Global Distribution of Microbial Abundance and Biomass in Subseafloor Sediment. Proc. Natl. Acad. Sci. USA.

[2] Jørgensen B.B., Boetius A. (2007). Feast and Famine—Microbial Life in the Deep-Sea Bed. Nat. Rev. Microbiol..

[3] Shu W.-S., Huang L.-N. (2022). Microbial Diversity in Extreme Environments. Nat. Rev. Microbiol..

[4] Rothschild L.J., Mancinelli R.L. (2001). Life in Extreme Environments. Nature.

[5] Di Lorenzo F., Billod J., Martín-Santamaría S., Silipo A., Molinaro A. (2017). Gram-Negative Extremophile Lipopolysaccharides: Promising Source of Inspiration for a New Generation of Endotoxin Antagonists. Eur. J. Org. Chem..

[6] Di Lorenzo F., Duda K.A., Lanzetta R., Silipo A., De Castro C., Molinaro A. (2022). A Journey from Structure to Function of Bacterial Lipopolysaccharides. Chem. Rev..

[7] Raetz C.R.H., Whitfield C. (2002). Lipopolysaccharide Endotoxins. Annu. Rev. Biochem..

[8] Pither M.D., Sun M.-L., Speciale I., Silipo A., Zhang Y.-Z., Molinaro A., Di Lorenzo F. (2022). Structural Determination of the Lipid A from the Deep-Sea Bacterium Zunongwangia Profunda SM-A87: A Small-Scale Approach. Glycoconj. J..

[9] Di Lorenzo F., Palmigiano A., Paciello I., Pallach M., Garozzo D., Bernardini M.-L., La Cono V., Yakimov M.M., Molinaro A., Silipo A. (2017). The Deep-Sea Polyextremophile Halobacteroides Lacunaris TB21 Rough-Type LPS: Structure and Inhibitory Activity towards Toxic LPS. Mar. Drugs.

[10] Kokoulin M.S., Dmitrenok P.S., Romanenko L.A. (2022). Structure of the Lipooligosaccharide from the Deep-Sea Marine Bacterium Idiomarina Zobellii KMM 231T, Isolated at a Depth of 4000 Meters. Mar. Drugs.

[11] Shimazu R., Akashi S., Ogata H., Nagai Y., Fukudome K., Miyake K., Kimoto M. (1999). MD-2, a Molecule That Confers Lipopolysaccharide Responsiveness on Toll-like Receptor 4. J. Exp. Med..

[12] Poltorak A., He X., Smirnova I., Liu M.-Y., Van Huffel C., Du X., Birdwell D., Alejos E., Silva M., Galanos C. (1998). Defective LPS Signaling in C3H/HeJ and C57BL/10ScCr Mice: Mutations in Tlr4 Gene. Science.

[13] Medzhitov R., Preston-Hurlburt P., Janeway C.A. (1997). A Human Homologue of the Drosophila Toll Protein Signals Activation of Adaptive Immunity. Nature.

[14] Garcia-Vello P., Di Lorenzo F., Zucchetta D., Zamyatina A., De Castro C., Molinaro A. (2022). Lipopolysaccharide Lipid A: A Promising Molecule for New Immunity-Based Therapies and Antibiotics. Pharmacol. Ther..

[15] Molinaro A., Holst O., Lorenzo F.D., Callaghan M., Nurisso A., D’Errico G., Zamyatina A., Peri F., Berisio R., Jerala R. (2015). Chemistry of Lipid a: At the Heart of Innate Immunity. Chem.-Eur. J..

[16] De Chiara S., De Simone Carone L., Cirella R., Andretta E., Silipo A., Molinaro A., Mercogliano M., Di Lorenzo F. (2025). Beyond the Toll-Like Receptor 4. Structure-Dependent Lipopolysaccharide Recognition Systems: How Far Are We?. ChemMedChem.

[17] Borio A., Holgado A., Passegger C., Strobl H., Beyaert R., Heine H., Zamyatina A. (2023). Exploring Species-Specificity in TLR4/MD-2 Inhibition with Amphiphilic Lipid A Mimicking Glycolipids. Molecules.

[18] De Chiara S., Olmeo F., Andretta E., De Simone Carone L., Mercogliano M., Belova V.S., Romanenko L.A., Kokoulin M.S., Silipo A., Molinaro A. (2025). Signals from the Sea: The Structural Peculiarity of Lipid A and Weak Immunostimulatory Lipopolysaccharide from Rheinheimera Japonica. RSC Chem. Biol..

[19] Romanenko L.A., Uchino M., Falsen E., Zhukova N.V., Mikhailov V.V., Uchimura T. (2003). Rheinheimera Pacifica Sp. Nov., a Novel Halotolerant Bacterium Isolated from Deep Sea Water of the Pacific. Int. J. Syst. Evol. Microbiol..

[20] Komandrova N.A., Kokoulin M.S., Kalinovsky A.I., Tomshich S.V., Romanenko L.A., Vaskovsky V.E. (2014). The O-Specific Polysaccharide of the Marine Bacterium Rheinheimera Pacifica KMM 1406T Containing d- and l-2-Acetamido-2-Deoxy-Galacturonic Acids. Carbohydr. Res..

[21] Casillo A., Lanzetta R., Parrilli M., Corsaro M.M. (2018). Exopolysaccharides from Marine and Marine Extremophilic Bacteria: Structures, Properties, Ecological Roles and Applications. Mar. Drugs.

[22] Galanos C., Lüderitz O., Westphal O. (1969). A New Method for the Extraction of R Lipopolysaccharides. Eur. J. Biochem..

[23] Westphal O., Jann K. (1965). Bacterial Lipopolysaccharides Extraction with Phenol-Water and Further Applications of the Procedure. Methods Carbohydr. Chem..

[24] Lenter M., Jann B., Jann K. (1990). Structure of the K16 Antigen from Escherichia Coli O7:K16:H-, A Kdo-Containing Capsular Polysaccharide. Carbohydr. Res..

[25] Bock K., Pedersen C., Tipson R.S., Horton D. (1983). Carbon-13 Nuclear Magnetic Resonance Spectroscopy of Monosaccharides. Advances in Carbohydrate Chemistry and Biochemistry.

[26] Costello C.E., Vath J.E., McCloskey J.A. (1990). Tandem Mass Spectrometry of Glycolipids. Methods in Enzymology.

[27] Barrau C., Di Lorenzo F., Menes R.J., Lanzetta R., Molinaro A., Silipo A. (2018). The Structure of the Lipid A from the Halophilic Bacterium Spiribacter Salinus M19-40T. Mar. Drugs.

[28] Silipo A., Lanzetta R., Amoresano A., Parrilli M., Molinaro A. (2002). Ammonium Hydroxide Hydrolysis. J. Lipid Res..

[29] Kokoulin M.S., Sokolova E.V., Elkin Y.N., Romanenko L.A., Mikhailov V.V., Komandrova N.A. (2017). Partial Structure and Immunological Properties of Lipopolysaccharide from Marine-Derived Pseudomonas Stutzeri KMM 226. Antonie Van. Leeuwenhoek.

[30] Di Lorenzo F. (2017). The Lipopolysaccharide Lipid A Structure from the Marine Sponge-Associated Bacterium Pseudoalteromonas Sp. 2A. Antonie Van. Leeuwenhoek.

[31] Pallach M., Di Lorenzo F., Duda K.A., Le Pennec G., Molinaro A., Silipo A. (2019). The Lipid A Structure from the Marine Sponge Symbiont Endozoicomonas Sp. *HEX* 311. ChemBioChem.

[32] Cirella R., Andretta E., De Simone Carone L., Olmeo F., Sun M., Zhang Y., Mercogliano M., Molinaro A., Silipo A., Di Lorenzo F. (2025). Cold-Adapted Lipid A from Polaribacter Sp. *SM*1127: A Study of Structural Heterogeneity and Immunostimulatory Properties. ChemBioChem.

[33] Mercogliano M., De Chiara S., De Nicola A., Cardellini J., Montis C., Yakimov M.M., La Cono V., Crisafi F., Silipo A., Berti D. (2024). Bucking the Trend: Understanding Lipopolysaccharide Structure and Outer Membrane Dynamics in Cold-Adapted Pseudomonas Isolated from Enigma Lake, Antarctica. Chem. Sci..

[34] Di Guida R., Casillo A., Yokoyama F., Kawamoto J., Kurihara T., Corsaro M.M. (2020). Detailed Structural Characterization of the Lipooligosaccharide from the Extracellular Membrane Vesicles of Shewanella Vesiculosa HM13. Mar. Drugs.

[35] Di Lorenzo F., Crisafi F., La Cono V., Yakimov M.M., Molinaro A., Silipo A. (2020). The Structure of the Lipid A of Gram-Negative Cold-Adapted Bacteria Isolated from Antarctic Environments. Mar. Drugs.

[36] Di Lorenzo F., Paciello I., Fazio L.L., Albuquerque L., Sturiale L., da Costa M.S., Lanzetta R., Parrilli M., Garozzo D., Bernardini M.L. (2014). Thermophiles as Potential Source of Novel Endotoxin Antagonists: The Full Structure and Bioactivity of TheLipo-oligosaccharide from Thermomonas Hydrothermalis. ChemBioChem.

[37] Silipo A., Di Lorenzo F., Fazio L.L., Paciello I., Sturiale L., Palmigiano A., Parrilli M., Grant W.D., Garozzo D., Lanzetta R. (2013). Structure and Immunological Activity of the Lipopolysaccharide Isolated from the Species Alkalimonas Delamerensis. Eur. J. Org. Chem..

[38] Carillo S., Pieretti G., Lindner B., Romano I., Nicolaus B., Lanzetta R., Parrilli M., Corsaro M.M. (2013). The Lipid A from the Haloalkaliphilic Bacterium Salinivibrio Sharmensis Strain BAGT. Mar. Drugs.

[39] Pither M.D., Mantova G., Scaglione E., Pagliuca C., Colicchio R., Vitiello M., Chernikov O.V., Hua K.-F., Kokoulin M.S., Silipo A. (2021). The Unusual Lipid a Structure and Immunoinhibitory Activity of LPS from Marine Bacteria Echinicola Pacifica KMM 6172T and Echinicola Vietnamensis KMM 6221T. Microorganisms.

[40] Cirella R., Pagliuca C., Pither M.D., Scaglione E., Nedashkovskaya O.I., Chernikov O.V., Hua K., Colicchio R., Vitiello M., Kokoulin M.S. (2023). Pushing the Boundaries of Structural Heterogeneity with the Lipid A of Marine Bacteria Cellulophaga. ChemBioChem.

[41] Andretta E., De Chiara S., Pagliuca C., Cirella R., Scaglione E., Di Rosario M., Kokoulin M.S., Nedashkovskaya O.I., Silipo A., Salvatore P. (2024). Increasing Outer Membrane Complexity: The Case of the Lipopolysaccharide Lipid A from Marine Cellulophaga Pacifica. Glycoconj. J..

[42] Casillo A., Parrilli E., Tutino M.L., Corsaro M.M. (2019). The Outer Membrane Glycolipids of Bacteria from Cold Environments: Isolation, Characterization, and Biological Activity. FEMS Microbiol. Ecol..

[43] Filshtein A.P., Belova V.S., Kuzmich A.S., Romanenko L.A., Kokoulin M.S. (2025). Structural and Immunological Insights into the Lipooligosaccharide of the Marine Bacterium Kangiella Japonica KMM 3897. Mar. Drugs.

[44] Di Guida R., Casillo A., Stellavato A., Di Meo C., Kawai S., Kawamoto J., Ogawa T., Kurihara T., Schiraldi C., Corsaro M.M. (2021). Complete Lipooligosaccharide Structure from Pseudoalteromonas Nigrifaciens Sq02-Rifr and Study of Its Immunomodulatory Activity. Mar. Drugs.

[45] Casillo A., Parrilli E., Filomena S., Lindner B., Lanzetta R., Parrilli M., Tutino M.L., Corsaro M.M. (2015). Structural Investigation of the Oligosaccharide Portion Isolated from the Lipooligosaccharide of the Permafrost Psychrophile Psychrobacter Arcticus 273-4. Mar. Drugs.

[46] Ierano T., Silipo A., Nazarenko E.L., Gorshkova R.P., Ivanova E.P., Garozzo D., Sturiale L., Lanzetta R., Parrilli M., Molinaro A. (2010). Against the Rules: A Marine Bacterium, Loktanella Rosea, Possesses a Unique Lipopolysaccharide. Glycobiology.

[47] Komandrova N.A., Kokoulin M.S., Isakov V.V., Tomshich S.V., Romanenko L.A. (2012). Structure of the O-Specific Polysaccharide from a Marine Bacterium Oceanisphaera Litoralis KMM 3654 T Containing ManNAcA. Carbohydr. Res..

[48] Laemmli U.K. (1970). Cleavage of Structural Proteins during the Assembly of the Head of Bacteriophage T4. Nature.

[49] Tsai C.-M., Frasch C.E. (1982). A Sensitive Silver Stain for Detecting Lipopolysaccharides in Polyacrylamide Gels. Anal. Biochem..

[50] Di Lorenzo F., Palmigiano A., Albitar-Nehme S., Pallach M., Kokoulin M., Komandrova N., Romanenko L., Bernardini M.L., Garozzo D., Molinaro A. (2018). Lipid A Structure and Immunoinhibitory Effect of the Marine Bacterium Cobetia Pacifica KMM 3879T. Eur. J. Org. Chem..

[51] Gerwig G.J., Kamerling J.P., Vliegenthart J.F.G. (1979). Determination of the Absolute Configuration of Monosaccharides in Complex Carbohydrates by Capillary g.l.c. Carbohydr. Res..

