# Supplementary material for: Structural Elucidation of the Lipooligosaccharide from the Deep-Sea Bacterium Rheinheimera pacifica KMM 1406T
Source: Mar Drugs. 2026 Sep 15;24(9):322. doi: 10.3390/md24090322 (PMC13608308; doi:10.3390/md24090322)
Supplement: Supplementary file 1 [file marinedrugs-24-00322-s001.zip › marinedrugs-4546586-supplementary.pdf]

Structural Elucidation of the Lipooligosaccharide from the Deep-Sea Bacterium *Rheinheimera pacifica* KMM 1406^T

Vlada S. Belova †, Lyudmila A. Romanenko, Pavel S. Dmitrenok and Maxim S. Kokoulin *,†

G.B. Elyakov Pacific Institute of Bioorganic Chemistry, Far Eastern Branch, Russian Academy of Sciences, 159/2, Prospect 100 let Vladivostoku, Vladivostok, 690022, Russia; vladabelova306@gmail.com; lro@piboc.dvo.ru; paveldmt@piboc.dvo.ru; maxchem@mail.ru

* Correspondence: maxchem@mail.ru

† These authors contributed equally to this work

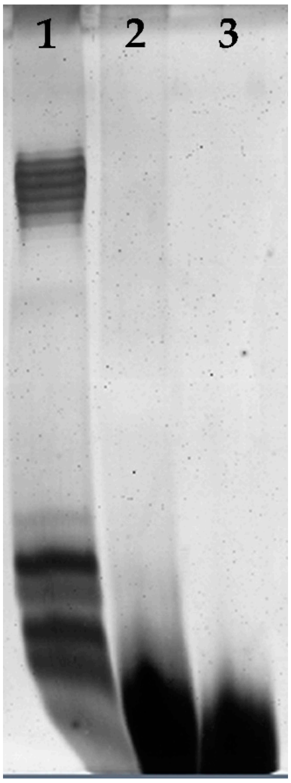

Figure S1. Silver-stained electrophoregram: lane 1 - *E. coli* 0111:B4 LPS 5 µg, lane 2 – *R. pacifica* KMM 1406^T 8 µg, lane 3 – *R. pacifica* KMM 1406^T 4 µg

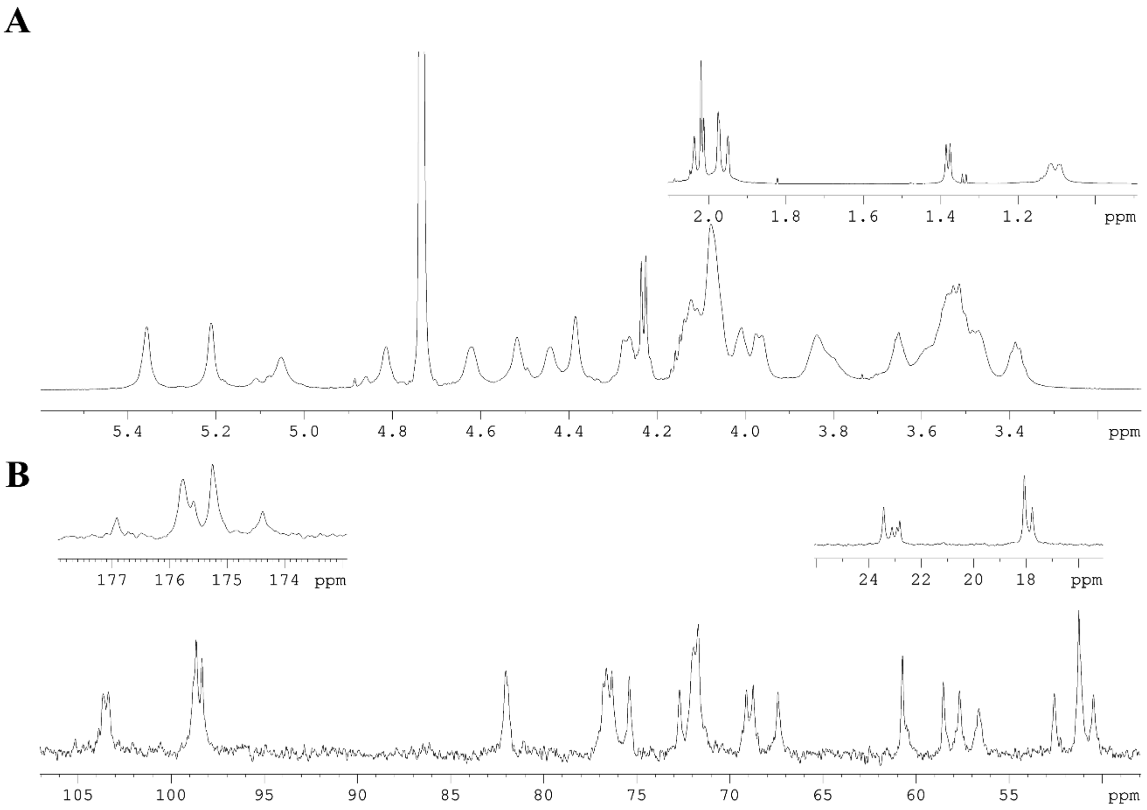

Figure S2. ^{1}H (**A**) and ^{13}C NMR (**B**) spectra of the CPS from *R. pacifica* KMM 1406^T

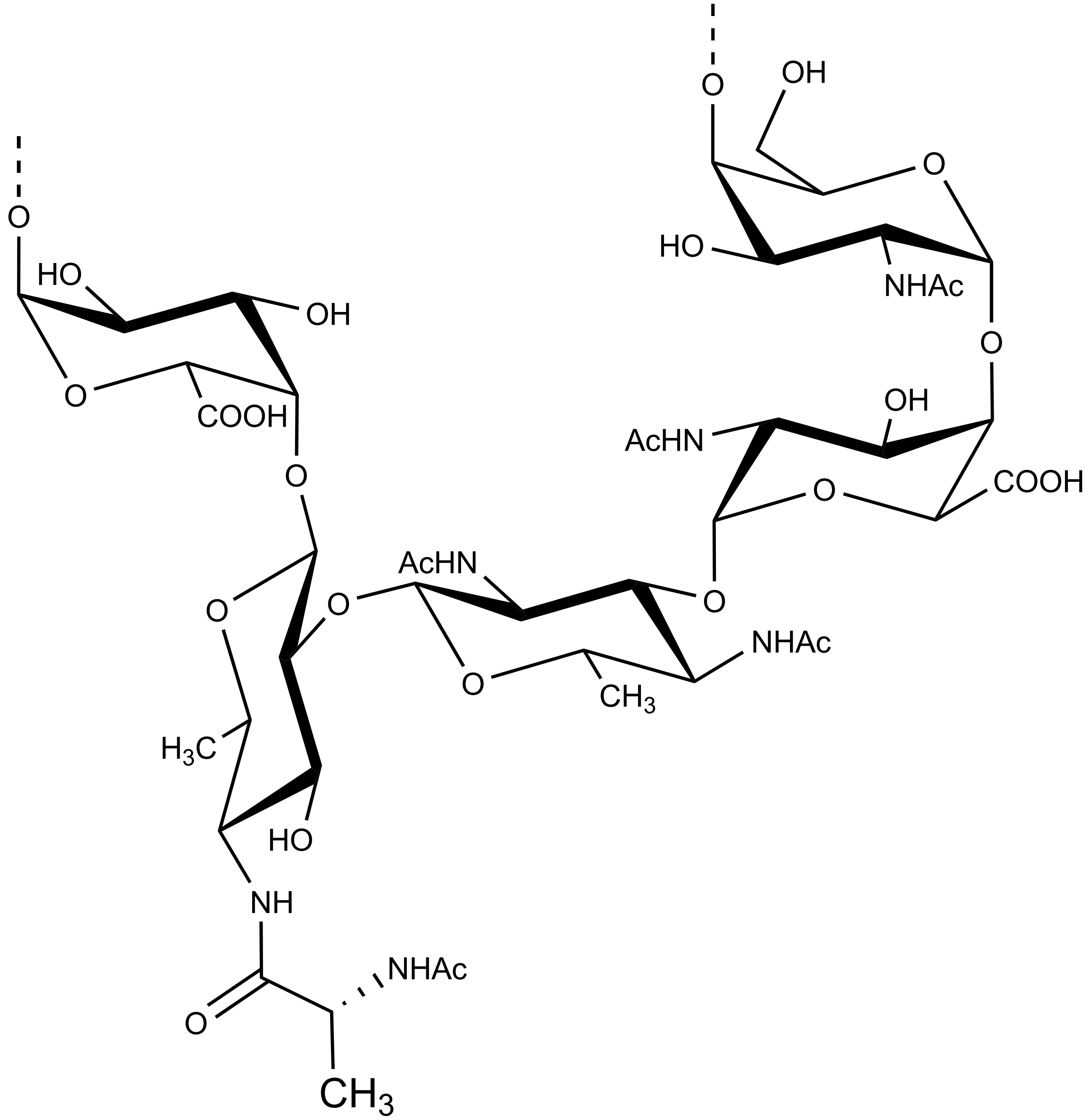

Figure S3. The structure of the CPS from *R. pacifica* KMM 1406T
